# Determinants of Intimate Partner Violence against Pregnant Women in Ethiopia: A Systematic Review and Meta-Analysis

**DOI:** 10.1155/2022/4641343

**Published:** 2022-03-26

**Authors:** Berhanu Boru Bifftu, Yonas Deressa Guracho

**Affiliations:** ^1^University of Gondar College of Medicine and Health Science, School of Nursing, Gondar, Ethiopia; ^2^Bahar Dar University, College of Medicine and Health Science, Department of Psychiatry, Ethiopia

## Abstract

**Background:**

Intimate partner violence (IPV) against pregnant women is a recognized global public health problem affecting all spheres of women and unborn infants. In Ethiopia, although inconsistent, individual studies avail; there is a dearth of systematic reviews and meta-analysis about the prevalence and associated factors of intimate partner violence. Thus, the present study was aimed at determining the pooled prevalence of IPV and its determinant factors during pregnancy.

**Methods:**

The report of meta-analysis follows the Preferred Reporting Items for Systematic Review and Meta-Analysis 20 guideline (PRISMA 20). Databases including PubMed/Medline, CINAHL, SCOPUS, HINARI (research4life), AJOL, IRIS, and AIM were searched. Heterogeneity test was assessed by the Cochrane chi-square (*χ*2) and quantified by *I*^2^ statistics test. Publication bias was tested by funnel plots and Egger's test. Sensitivity test and subgroup analysis were also performed. Effect size was calculated by random effects model.

**Results:**

A total of 26 studies, including data from 13, 912 participants, were included in the analysis. The prevalence of IPV ranged from 7% to 81% with overall estimated pooled prevalence of 37% (30% -44%, *I*^2^ = 96.5%, *p* ≤ 0.001). Of this, the prevalence of physical, sexual, and psychological violence was 24% (95% CI; 19%-30%), 21% (95% CI; 16%-26%), and 27% (95% CI; 22%-32%), respectively. Factors such as lack of formal education, childhood violence, rural residency, low decision-making power, family history of violence, attitude, unplanned and unwanted pregnancy by women and partners, late initiation of antenatal care, partner alcohol, and khat use were associated with IPV.

**Conclusion:**

More than one-third of pregnant women experienced IPV. The most prevalent form of IPV was psychological violence followed by physical and sexual violence. The identified risk for IPV including victim, pregnancy, and perpetrator-related factors indicated the need of a holistic approach in the promotion, prevention, and treatment of IPV. The finding of this study suggests the need of strengthening women empowerments (capacity building) against traditional beliefs, attitudes, and practices. This study also suggests the need of evaluation and strengthening the collaborative work among different sectors such as policy-makers, service providers, administrative personnel, and community leaders, including the engagement of men partners.

## 1. Background

Intimate partner violence (IPV) against pregnant women is a recognized global public health problem [1, 2] affecting psychological, social, physical, and reproductive health including the following: unintended pregnancy, induced abortion, bleeding, HIV, and other sexual transmitted infections [1–5]. Moreover, IPV affects the life of unborn infants by damaging the placenta, rupture of the uterus, fetal trauma [2, 6, 7], spontaneous abortion, preterm labor, preterm delivery, and low birth weight [4, 8, 9].

Globally, one in every three women experienced IPV at some point in their life time [6]. In Africa, the prevalence of IPV was found to be 37% [6]. In Ethiopia, the prevalence of IPV was ranging from 50% to 81% [1, 4, 7–13]. The high proportion of IPV in Ethiopia was associated with the deep-rooted perception of the communities' and women's acceptance of IPV [14]. That is why up to 93% of women who have experienced IPV were not disclosed to anyone [2, 12, 13, 15–17]. According to the 2016 Ethiopian Demographic and Health Survey (EDHS2016), out of 34% of women who have experienced IPV, 66% of them were not told to any one [18]. During pregnancy, the prevalence of physical, sexual, psychological, and overall prevalence of IPV was ranging from 2% to 35%, 9% to 40%, 22% to 65% [19], and 0.9% to 57% [5, 20, 21], respectively.

Although many of the risk factors were similar among pregnant and nonpregnant women, various studies have shown that pregnancy could be one of the risk factors for the initiation or escalation of IPV for certain women. Generally, factors including (i) pregnancy-related such as unplanned pregnancy, unwanted pregnancies, and elective termination of pregnancy; (ii) victim-related characteristics such as age, marital status, ethnicity, education, employment, substance use; and (iii) perpetrator-related characteristics such as dependency, jealousy, and possessiveness towards female partner were factors associated with IPV during pregnancy [5, 20, 21]. In spite of the high prevalence of IPV and its associated adverse impact, there is a dearth of epidemiological evidence in Ethiopia and only a systematic review and meta-analysis were reported from eight studies [22]. Since, the publications of this systematic review and meta-analysis (2018), several studies have been published. Therefore, the current study could benefit the field by addressing the identified gap such as limited number of studies (8 studies), survey period, study sites, and the inconsistent individual reports with ranging prevalence of 7% to 81% [4, 7, 23–46]. This discrepancy between studies could be due to the variation in survey periods, sampling, study sites, diagnostic criteria, and demographic characteristics. Evidence-based strategies such as systematic reviews and meta-analyses are ideal to address such issues. Therefore, the present study was aimed at determining the estimate pooled prevalence of IPV and associated factors against pregnant women in Ethiopia.

## 2. Methods

This systematic review and meta-analysis followed the Preferred Reporting Items for Systematic Review and Meta-Analysis 20 (PRISMA 20) [47].

### 2.1. Search Strategy

Initially, the Database of Abstracts of Reviews of Effects (DARE) and the Cochrane Database of Systematic Reviews (CDSR) were searched to confirm the absence of similar studies in Ethiopia.

Then, the first search strategy was started using a compressive search of electronic databases including the following: PubMed/Medline, CINAHL, Scopus, HINARI (research4life), AJOL, IRIS, and AIM. Using the following key words: epidemiology, prevalence, incidence, gender based violence, domestic violence, intimate partner violence, spouses violence, physical abuse, physical violence, emotions violence, emotions abuse, psychological violence, psychological abuse, sex violence, sex abuse, sexual coercion, rape, associated, risk, pregnancy, pregnant, maternal, pregnant mothers, antenatal mothers, pregnant women, antenatal, during pregnancy, and Ethiopia; Medical Subject Headings (MeSH) terms and Boolean operators (“AND” and/“OR”) were established for each databases. A combination of MeSH thesaurus and text words combined with appropriate Boolean operators (AND and OR) was formulated for PubMed/Medline (full search query for the database PubMed/MEDLINE at annexed). Similarly, CINAHL, Scopus, HINARI (research4life), IRIS, AJOL, and AIM were searched using search terms tailored to each database. The second stage of the search phase involved scanning relevant websites, including national and international institutions and research centers, and web of science (Google and Google Scholars) for grey literatures. Finally, the reference lists of the included articles were searched. All searches were performed until January, 07, 2021.

### 2.2. Selection of Studies

All retrieved studies were imported in EndNote X7 (Thomson Reuters, New York, USA) and duplicates were removed. The study selection process had two stages. The first stage was screening the title and abstract of the retrieved studies. The second stage was a perusal of their full text to determine eligibility. Two reviewers (YDG and BBB) independently screened the studies. Disagreements were solved by discussion.

## 3. Definition of Concepts

In this study, IPV was defined as any violence whether physical, psychological, and sexual, or any combination of them, regardless of the legal status of the relationship. Physical violence is defined as one or more intentional acts of physical aggression such as pushing, slapping, throwing, hair pulling, punching, hitting, kicking, or burning, perpetrated with the potential to cause harm, injury, or death. Psychological violence is defined as one or more acts or threats of acts including shouting, controlling, intimidating, humiliating, and threatening the victim [1, 2]. Sexual violence is defined as the use of force, coercion, or psychological intimidation to force woman to engage in a sex act against her will, whether or not it is completed [1, 2, 48].

### 3.1. Eligibility Criteria

Participants: this review targeted studies that were carried out during pregnancy

Outcome measure: this systematic review and meta-analysis has two main outcomes which are the (i) estimated pooled prevalence of IPV and (ii) factors associated with IPV

Study design: observational studies (cross-sectional and cohort/longitudinal)

Setting: this review included studies that were carried out in Ethiopia.

Studies that focused on case reports, conference, and abstracts were excluded.

### 3.2. Data Extraction

Data were extracted from the eligible studies using a preconceived and piloted data extraction Microsoft Excel spreadsheet. Data were simultaneously extracted by two independent reviewers (BBB and YDG). The extracted data items include the following: name of the first author, year of publication, study setting/region, study design, data collection tool, sample size, and number of cases/prevalence. For the analysis of associated factors, adjusted odds ratio at 95% confidence interval was extracted to address confounders.

### 3.3. Quality Assessment

The Newcastle-Ottawa quality assessment tool, adapted for cross-sectional studies [49], was used for quality assessment. This tool has three main parts (selection, comparability, and outcome). The first part (selection) has five stars and assesses the methodological quality of the study. The second part of the tool evaluates the comparability of the study. The third part of the tool assesses the quality of the original article's outcome with respect to the statistical analysis. Individual paper was graded with a score ranging from zero to ten stars. The overall quality of each article was determined using the sum of each star of the three parts and defined as high quality for a score ≥ 6 out of 10, medium (fulfilling 50% of the quality assessment criteria), and poor for < 4.

### 3.4. Data Analysis

The extracted data were exported to Stata version 14. Metaprop command in Stata was used to calculate the pooled prevalence estimate of DV with 95% confidence intervals (CIs) using a random-effects model via the DerSimonian and Laird transformed inverse-variance method [50]. Heterogeneity was assessed using the Cochrane chi-square (*χ*^2^) and *I*^2^ statistics. *I*^2^ value greater than 50% was considered indicative of substantial heterogeneity [51]. Sensitivity analyses were carried out to assess the contribution of each individual study to the overall effect size. In addition to this, subgroup analyses were conducted by study region, sample size, quality of each study, and publication year. Furthermore, publication bias was tested by the funnel plot [52] and Egger's test [53]. A *p* value < 0.1 was considered indicative of statistically significant publication bias. The meta-analysis of associated factors was conducted when at least two studies reported the same associated factors based on the overall IPV [54]. Results were presented using texts, tables, forest plot, and summary of descriptive statistics.

## 4. Results

**Figure 1:**
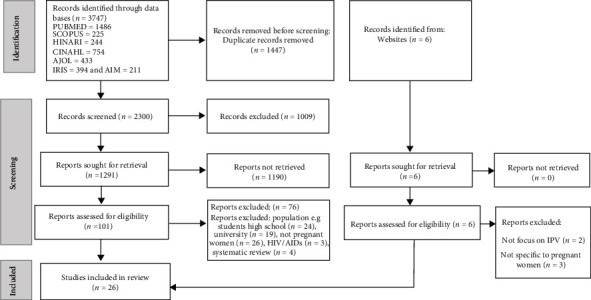
Flow diagram of included studies.

The initial database search resulted in 3747 publications. Of these records, 2327/1474 were excluded because of duplication. Of the remaining 2300, 1009 studies were excluded as their title and abstract reading were not related to the outcome. From the remaining 1291 studies, 1190 articles were excluded based on the full text/information and found unclear results. Again, from the remaining 101 studies, 76 publications were excluded based on the eligibility criteria. Moreover, from the other source, 6 studies were located and following the removal of 5 studies because the focus were not pregnant women IPV. The remaining 26 studies were included in the systematic review and meta-analysis (Figure 1).

### 4.1. Study Characteristics

In this study (detail Table 1), a total of 26 studies, including data from 13, 912 study participants, were included in the analysis for the overall IPV. Regarding the types of IPV, the most common type of reported IPV was physical violence (*n* = 24), followed by psychological (*n* = 21), and sexual violence (*n* = 19). Moreover, for the analysis of associated factors, a total of 17 studies, including data from 10, 940 participants, were included. These studies were carried out in five different regions: Southern Nation and Nationalities of People (*n* = 4), Amhara (*n* = 7), Oromia (*n* = 8), Addis Ababa (*n* = 3), and Tigray (*n* = 4). Majority (62%) of the included studies used institution-based cross-sectional study design. Eighty-five percent of the included studies used probability (e.g., random sampling) sampling, whereas 15% relied on nonprobability (convenience) sampling. Most (77%) studies were published between 2016 and 2020. The sample sizes of included studies ranged from 183 to 3015. For the assessment of IPV, the WHO questionnaires (*n* = 22) and AAS (*n* = 1) were used, whereas the remaining three studies used self-developed assessment tools.

### 4.2. Quality of Included Studies

The quality of the included studies was ranging from 4 to 9. Majority (88%) had good quality, while the remaining 12% had fair quality (Table 2).

### 4.3. Test of Heterogeneity, Sensitivity, Publication Bias, and Subgroup Analysis

**Figure 2:**
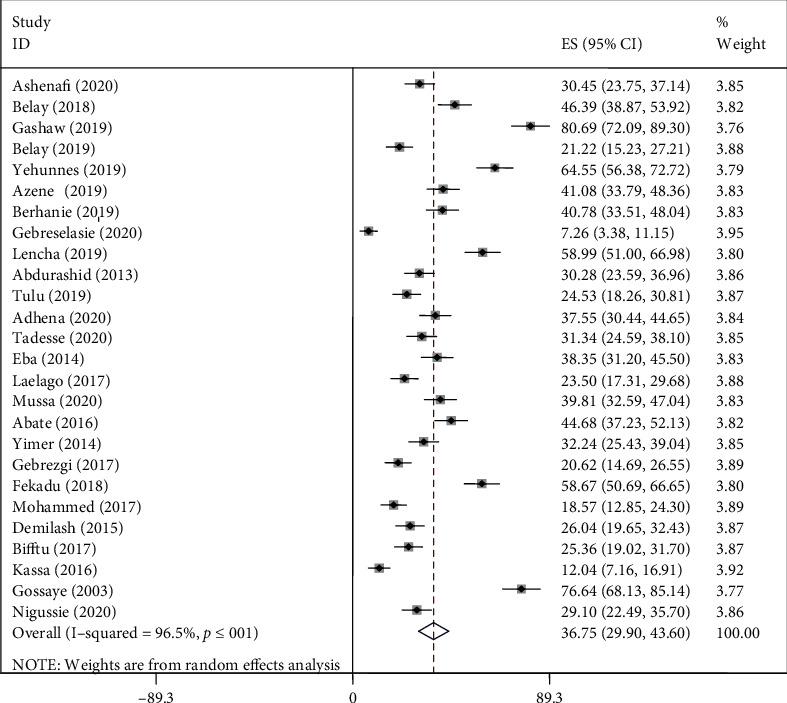
Pooled prevalence of overall IPV during pregnancy.

**Figure 3:**
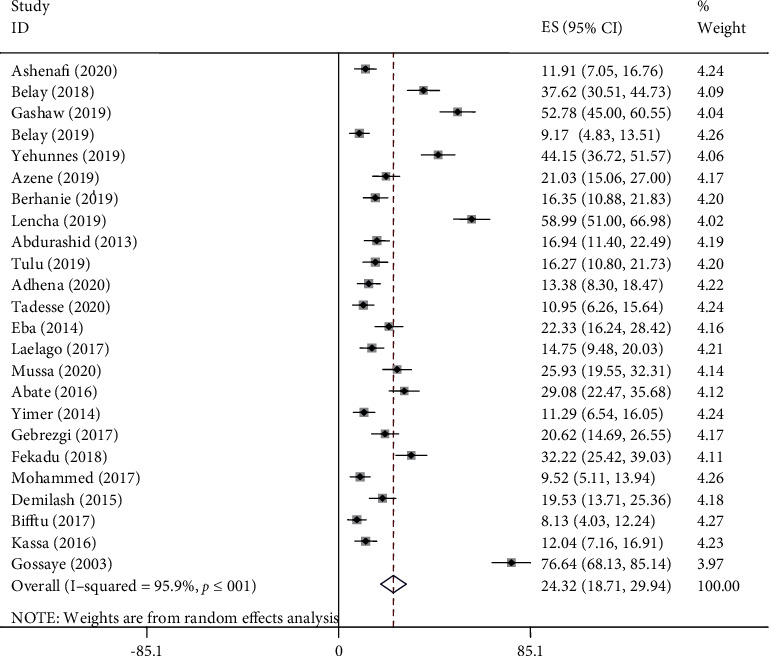
Pooled prevalence of physical violence during pregnancy.

**Figure 4:**
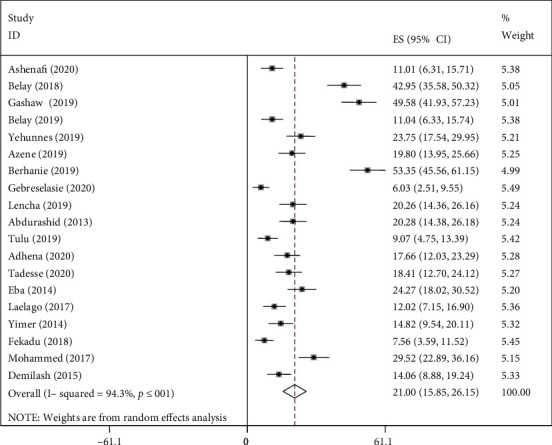
Pooled prevalence of sexual violence during pregnancy.

**Figure 5:**
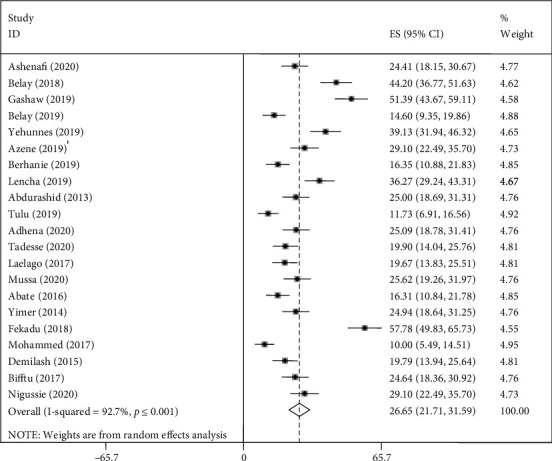
Pooled prevalence of psychological violence during pregnancy.

**Figure 6:**
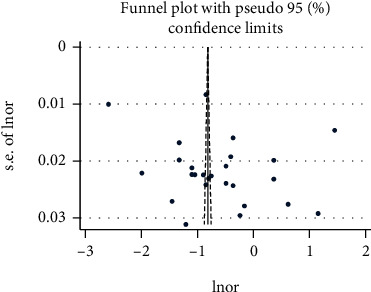
Funnel plot with pseudo 95 % confidence interval that investigated the heterogeneity of the pooled prevalence of overall IPV.

**Figure 7:**
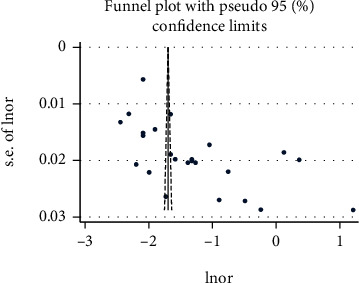
Funnel plot with pseudo 95 % confidence interval that investigated the heterogeneity of the pooled prevalence of physical violence.

**Figure 8:**
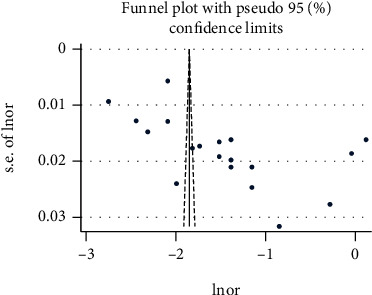
Funnel plot with pseudo 95 % confidence interval that investigated the heterogeneity of the pooled prevalence of sexual violence.

**Figure 9:**
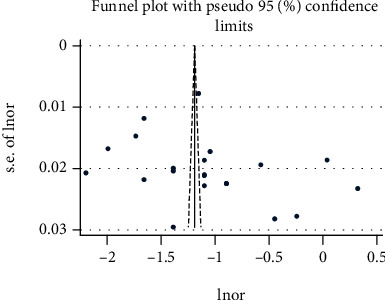
Funnel plot with pseudo 95 % confidence interval that investigated the heterogeneity of the pooled prevalence of psychological violence.

We found evidence of significant heterogeneity for the overall estimate pooled prevalence of IPV (*I*^2^ = 96.5% and *p* ≤ 0.001), physical violence (*I*^2^ = 95.9% and *p* ≤ 0.001), sexual violence (*I*^2^ = 94.3% and *p* ≤ 0.001), and psychological violence (*I*^2^ = 92.7% and *p* ≤ 0.001) (Figures 2, 3, 4, and 5, respectively); yet, the sensitivity analysis showed that none of the point estimates was outside of the overall 95% confidence. The subgroup analyses of overall IPV by study setting, study design, year of publication, sample size, and quality of study were not identified the source of heterogeneity for the overall IPV; yet, the highest pooled prevalence was reported from studies carried out in Oromia region (50.7% (95% CI: 35%-66%). However, the subgroup analysis of physical violence indicated heterogeneity by the study setting (Tigray: 17% (95% CI: 12%-21%, *I*^2^ = 39%, and *p* = 0.193)). Moreover, the subgroup analysis of sexual violence by study setting (SNNP: 12% (95% CI: 8%-15%, *I*^2^ = 0%, and *p* = 0.775)) and study design (community based 12% (95% CI: 9%-15%, *I*^2^ = 0%, and *p* = 0.491) also indicated as source of heterogeneity. Moreover, the subgroup analysis of psychological violence by year of publication (2003-2015 : 25% (95% CI: 21%-28%, *I*^2^ = 31.69%, and *p* = 0.223)) could be a source of heterogeneity (Table 3). Regarding the publication bias test, there was no evidence of publication bias from the visual inspection of the funnel plot and Egger's test for the overall IPV [(Figure 6) and Egger's test (*p* = 0.111)], sexual violence [(Figure 7) and Egger's test (*p* = 0.068)], psychological violence [(Figure 8) and Egger's test (*p* = 0.255)], and physical violence [(Figure 9) and Egger's test (*p* = 0.442)].

### 4.4. Prevalence of Intimate Partner Violence

In this systematic review, the lowest (7%) and the highest (81%) prevalence of IPV were reported from Tigray and Oromia Region, respectively. The prevalence of IPV ranged from 7% to 81% with overall estimated pooled prevalence of 37% (30% -44%, *I*^2^ = 96.5%, and *p* ≤ 0.001). Of this, the prevalence of physical, sexual, and psychological violence was 24% (95% CI; 19%-30%), 21% (95% CI; 16%-26%), and 27% (95% CI; 22%-32%), respectively.

### 4.5. Factors Associated with Intimate Partner Violence during Pregnancy

A total of 17 studies, including 53 distinct odds ratios, from 13 unique risk factors were included in the meta-analysis. Based on previous similar studies, these factors were broadly categorized as follows: (i) victim-related: lack of formal education, history of childhood violence, rural residency, lack of decision-making power, family history of violence, and attitude toward IPV; (ii) pregnancy-related factors: unplanned pregnancy, unwanted pregnancy, and late initiation of ANC; and (iii) perpetrator-related factors: lack of formal education, partner alcohol use, partner khat chewing, and unwanted pregnancy (Table 4).

#### 4.5.1. Victim Related

In this study, the pooled effect of five studies showed that those participants who have no formal education were around four times more likely experienced IPV compared to those pregnant women who have formal education (AOR = 3.88; 95% CI: 1.48, 6.27, *I*^2^ = 0.0, and *p* = 0.638). Similarly, those participants who had history of childhood violence (AOR = 3.14; 95% CI: 1.37, 4.90, *I*^2^ = 88.8, and *p* = 0.003), family history of violence (AOR = 1.68; 95% CI: 1.14, 2.22, *I*^2^ = 0, and *p* = 0.650), attitude toward DV (AOR = 12.92; 95% CI: 6.58, 19.25, *I*^2^ = 0, and *p* = 0.835), and rural residency (AOR = 2.48; 95% CI: 1.29, 3.67, *I*^2^ = 0, and *p* = 0.638) were associated with DV. On the other hand, the pooled effect of four studies showed that those women's who had no decision making powers were more than 1.3 time (AOR = 1.30; 95% CI: 1.03, 1.58, *I*^2^ = 87.3, and *p* ≤ 0.001) more likely experienced IPV as compared to those who had decision making power; yet, insignificant.

#### 4.5.2. Pregnancy-Related Factors

Unplanned pregnancy (AOR = 1.77; 95% CI: 1.48, 2.05, *I*^2^ = 24.9, and *p* = 0.255), unwanted pregnancy (AOR = 1.54; 95% CI: 1.00, 2.08, *I*^2^ = 70, and *p* = 0.019), and late initiation of ANC (AOR = 1.30; 95% CI: 1.15, 1.44, *I*^2^ = 0, and *p* = 0.374) were pregnancy-related factors associated with pregnant women's experience of IPV.

#### 4.5.3. Perpetrator-Related Characteristics

In this study, the pooled effect of eleven studies showed that partner alcohol use was two times (AOR = 2.19; 95% CI: 1.66, 2.71, *I*^2^ = 54.9, and *p* = 0.014) more likely experienced IPV compared to those who did not use alcohol. The result of publication bias from the visually examined funnel plot and Egger's tests showed that there was no evidence of small study effect using Egger's test (*p* = 0.461). The pooled effect of the three studies showed that partner khat use was around two times (AOR = 1.73; 95% CI: 1.42, 2.05, *I*^2^ = 0, and *p* = 0.593) more likely experienced IPV compared to those partners who did not chew khat. The pooled effect of two studies showed that partner's unwanted pregnancy was 1.2 times (AOR = 1.20; 95% CI: 1.03, 1.36, *I*^2^ = 79.4, and *p* = 0.027) more likely experienced IPV. On the other hand, the pooled effect of six studies showed that the partner with no formal education was 1.1 times (AOR = 1.10; 95% CI: 0.93, 1.28, *I*^2^ = 76.6, and *p* = 0.013) experienced IPV compared to those partner who have formal education, yet statistically not significant.

## 5. Discussion

In this systematic review and meta-analysis, more than one in three (37%) pregnant women experienced IPV. The overall pooled prevalence of psychological violence [27% (95% CI; 22%-32%)] was higher than physical [24% (95% CI; 19%-30%)] and sexual violence [21% (95% CI; 16%-26%)]. Comparing with other studies, the observed range of IPV (7% to 81%) in this systematic review and meta-analysis is consistent with previous systematic review and meta-analysis conducted in Ethiopia (12% to 45%) [23], Nigeria (2.3% to 44.6%) [21], African countries (0.9% to 57%) [5, 19, 21], and the WHO Multi-Country Study (1% to 28%) [55].

The estimated pooled prevalence of IPV (37%) in this study is higher than the reported prevalence of IPV in Ethiopia (26.1% (95% CI: 20, 32.3)), Africa (15%), China (7.7%) [56], and a meta-analysis of 92 studies across 23 countries [19.8% (13.3% for developed countries and 27.7% for developing countries [57]. On the other hand, lower than a systematic review and meta-analysis conducted in Iran (48% (95% CI: 38-58)) [58]. This variation may be attributed to the sociocultural difference of the study population, educational status, lack of access to correct information and reproductive health information, and stressful life events. This is supported by evidence from previous studies [5, 57] as factors such as unplanned pregnancy, low socioeconomic status, lower education status, stressful life events, and lack of social support were associated with IPV during pregnancy [5, 57, 58].

Regarding the different form of IPV, in this study, the overall pooled prevalence of psychological violence (27%), physical violence (24%), and sexual violence (21%) is similar with previous systematic review and meta-analysis carried out in Ethiopia with reported prevalence of 16%, 21%, and 16% for physical, psychological, and sexual violence, respectively [23]. Similarly findings from Iran revealed that the pooled prevalence of physical, psychosocial, and sexual violence was found to be 17% (95% CI: 12-32), 41% (95% CI: 33-50), and 21% (95% CI: 16-23), respectively [58]. Another systematic review and meta-analysis of 92 studies across 23 countries [psychological violence (28.4%), physical abuse (13.8%), and sexual abuse was 8%] [57]. Another systematic review and meta-analysis of 73 studies carried out across the worldwide showed that the pooled prevalence of sexual violence during pregnancy was 17% (CI 95%:15%-18%) [59]. In this study, the observed highest prevalence of psychological violence and lowest prevalence of sexual violence may be associated with traditional culture and women's unwilling to talk about sex-related topics and acceptance IPV. This is supported with other similar studies [15, 60–62].

Regarding the associated factors, the association of lack of formal education; history of childhood violence; rural residency; lack of decision-making power; family history of violence; attitudes toward unplanned pregnancy, unwanted pregnancy, and late initiation of ANC; partner alcohol use, partner khat chewing; and unwanted pregnancy with IPV supported previous meta-analysis [14, 59–62]. In Ethiopia, a previous systematic review and meta-analysis showed that women's educational status, partner's educational status, and partner's alcohol use were associated with IPV [22]. The findings of meta-analysis in Iran showed that low level of maternal education, partner use of tobacco, and regular visits of ANC were factors associated with IPV. Similarly, a systematic review and meta-analysis in China showed that number of children and unplanned pregnancies were factors significantly associated with IPV. A meta-analysis of 55 studies across the world showed that lower educational level, low socioeconomic status, single, partner alcohol use, unwanted pregnancy, and lifetime adversity were factors associated with IPV [57, 62].

Therefore, the finding of this study implies the following: high prevalence of IPV against pregnant women, which suggests the needs of strengthening the awareness of IPV and women's empowerment and evaluates the effectiveness of the national IPV prevention strategies. The identified risk for IPV including victim, pregnancy, and perpetrator-related factors indicated the need of a holistic approach in the promotion, prevention, and treatment of IPV. Evidence showed that victim-related interventions such as screening, in conjunction with supportive counseling, and strengthening women's empowerment; pregnancy-related interventions such as: strengthening community awareness toward family planning and perpetrator-related interventions such as: designing an effective controlling mechanisms for partner substance use were important in the control of IPV [5, 20, 21].

### 5.1. Strengths and Limitations of the Study

To manage heterogeneity bias and make the findings more meaningful, the use of a random effects model was to control the effects of heterogeneity, sensitivity analysis was to identify influential studies and subgroup analysis, and the funnel plot asymmetry and Egger test were used to help the findings become more meaningful. However, limitations like the use of different measurement tools (self-developed) may affect the different forms of domestic violence. The use of reference lists and Google Scholar to include the available studies may have overlooked some studies.

## 6. Conclusion

More than one-third of pregnant women experienced domestic violence. The most prevalent form of domestic violence was psychological violence followed by physical and sexual violence. The identified risk for IPV includes victim, pregnancy, and perpetrator-related factors which indicated the need of a holistic approach in the promotion, prevention, and treatment of IPV. The finding of this study suggests the need of strengthening women empowerments (capacity building) against traditional beliefs, attitudes, and practices. This study also suggests the need of evaluation and strengthening the collaborative work among different sectors such as policymakers, service providers, administrative personnel, and community leaders, including the engagement of men partners.

## Figures and Tables

**Table 1:** Characteristics of the included studies.

Author	Year	Design	Setting	Tool	Total	Prevalence of IPV number (percent)				Factors associated with IPV
						Overall	Physical	Sexual	Psychological	
Ashenafi	2020	CBCS	Oromia	WHO	3015	918 (30)	359 (12)	332 (11)	736 (24)	Age 20-30 (AOR = 1.56 (1.23, 1.98), partner khat use (AOR = 1.72 (1.41-2.11), late ANC(AOR = 1.25 (1.09-1.42), unwanted partner pregnancy (AOR = 1.19 (1.03-1.36), and no partner educational status (AOR = 1.26 (1.06-1.47)
Belay	2018	IBCS	Amhara	WHO	319	148 (46)	120 (38)	137 (43)	141 (44)	No formal education (APR = 7.0; 95% CI: 3.1, 17.8),age difference with partner > (APR = 2.9 (1.4, 5.9), decision by man (APR = 6.7; 95% CI: 2.3, 23.3), family arrangements of marriage (APR = 2.8; 95% CI: 1.2, 7.6), and no formal education of partner (APR = 2.8; 95% CI: 1.6, 4.8)
Gashaw	2019	IBCS	Oromia	AAS	720	581 (81)	380 (53)	357 (50)	370 (51)	Partner alcohol use (AOR = 9.3 (5.5,15.7), partner experienced violence as a child (AOR = 21.0 (13.1,33.4), partner witnessed violence as a child (AOR = 16.5 (10.5,26.0), woman childhood abused (AOR = 11.8 (7.3,19.2), family history of IPV (AOR = 12.5 (7.1,22.0),and decisionmaking by partner (AOR = 8.6 (5.9,12.6)
Belay	2019	CBCS	SNNP	WHO	589	125 (21)	54 (9)	65 (11)	86 (15)	Rural (AOR = 2.09; 95% CI = 1.06–4.09), family history of IPV (AOR = 14.00; 95% CI = 6.43–30.48), unwanted pregnancy (AOR = 9.64; 95% CI =3.44–27.03), husbands used alcohol (AOR = 17.08; 95% CI =3.83–76.19), depression (AOR = 4.71; 95% CI = 1.37–16.18), and low social support (AOR = 13.93; 95% CI = 6.98–27.77).
Yehunnes	2019	IBCS	Oromia	WHO	299	193 (65)	132 (44)	71 (24)	117 (31)	No formal education (OR = 6.3; 95% CI: 2.23, 11.65), husband's alcohol use (OR = 5.726; 95% CI 1.873, 11.51), husband history of arrest (OR = 2.59; 95% CI: 1.15, 5.88), and occupation of *husband (farmer)* (OR = 3.26; 95% CI: 1.29, 8.25)
Azene	2019	IBCS	Amhara	WHO	409	168 (41)	86 (21)	81 (20)	119 (29)	Lower educational status of partners (AOR = 3.26, 95% CI: 1.45–7.36), rural (AOR = 4.04, 95% CI: 1.17–13.93), partner alcohol use (AOR = 4.79, 95% CI: 2.08–11.04), early initiation of ANC (AOR = 0.44, 95% CI: 0.24–0.81)/0.56 (), age 17–26 years (AOR) = 0.21, 95% CI: 0.09–0.49), and choice of partner by the women only (AOR = 3.26,95% CI:1.24–8.57)
Berhanie	2019	IBCS	Tigray	SELF	954	389 (41)	156 (16)	509 (53)	156 (16)	—
Gebreselasie	2020	IBCS	Tigray	SELF	647	47 (7)	22 (3.4)	39 (6)	8 (1.2)	—
Lencha	2019	IBCS	Oromia	WHO	612	361 (59)	361 (59)	124 (20)	222 (36)	Partners alcohol use [AOR = 2.9; 95% CI: (1.5–5.4)], partners chewed khat [AOR = 1.7; 95% CI: (1.1–2.6)], partners smoked cigarette [AOR = 2.6; 95% CI: (1.4–4.9)], partners aggressive behavior [AOR = 2.8; 95% CI: (1.7–4.6)], partner age > / = 30 [AOR = 1.8; 95% CI: (1.2– 2.9)], unwanted pregnancy [AOR = 3.3; 95% CI: (1.9–5.5)], and history of adverse pregnancy outcome [AOR = 2.1; 95% CI: (1.2–3.6)]
Abdurashid	2013	IBCS	Addis Ababa	WHO	360	109 (30)	61 (17)	73 (20)	90 (25)	Unwanted pregnancy (AOR = 2.882 (1.693-4.903) and partner alcohol use(AOR = 1.763 (.848-3.666)
Tulu	2019	IBCS	Oromia	WHO	375	92 (25)	61 (16)	34 (9)	44 (12)	Partner alcohol use (AOR = 3.33, 95% CI: 1.22-9.11), unplanned pregnancy (AOR = 1.76, 95% CI:1.32-2.88), and unwanted pregnancy (AOR = 1.12, 95% CI: 1.06-2.28)
Adhena	2020	IBCS	Tigray	WHO	538	202 (36)	72 (13)	95 (17)	135 (25)	Unplanned pregnancy (AOR = 4.56, 95% CI: (2, 10.28)), unmarried women (AOR = 2.59, 95% CI: (1.18, 5.73), alcoholic partner (AOR = 3.3, 95% CI: (2.1, 5.16), spouse's multiple sexual partners (AOR = 5.1, 95% CI: (2.2, 12), acceptance of DV by women (AOR = 1.85, 95% CI: (1.1, 3.16)), low decision-making power (AOR = 2.64, 95% CI: (1.6, 4.3), and no interest in current pregnancy by partner (AOR = 5.9, 95% CI: (2.36, 14.9)
Tadesse	2020	IBCS	Amhara	WHO	402	126 (31)	44 (11)	74 (18)	80 (20)	
Eba	2014	IBCS	Addis Ababa	WHO	412	158 (38)	92 (22)	100 (24)	—	
Laelago	2017	IBCS	SNNP	WHO	183	43 (23)	27 (15)	22 (12)	36 (20)	Partners' alcohol use (AOR = 22 (7.4, 65.6), no formal education of the partners (AOR = 10.8 (1.06, 108.5), planned pregnancy (AOR = 0.23 (0.08, 0.67) OR 1.77 (1.23, 1.92, and low birth weight of the new born (AOR:14.3,95% CI: (5.03, 40.7).
Mussa	2020	IBCS	SNNP	WHO	648	258 (40)	168 (26)	24 (3.7)	166 (26)	Longer duration of marriage (AOR = 1.68, 95% CI: 1.01–2.79), unplanned pregnancy (AOR = 1.55, 95% CI: 1.03–2.34), controlling behaviour, (AOR = 2.23, 95% CI: 1.46–3.40), and attitude justifies DV (AOR = 1.60, 95% CI: 1.09–2.36)
Abate	2016	CBCS	Oromia	WHO	282	126 (45)	82 (29)	85 (30)	46 (16)	Lower educational status of partners (AOR 0.5, 95% CI 0.2, 0.9), dowry payment (AOR 8.7, 95% CI 4.2, 17.9), and no marriage ceremony (AOR 4.1, 95% CI 2, 8.2)
Yimer	2014	CBCS	Amhara	WHO	425	137 (32)	48 (11)	63 (15)	106 (25)	Childhood DV (AOR = 2.3, 95% CI 1.1–4.8), partner alcohol use (AOR = 3.4, 95% CI 1.6–7.4), and undesired pregnancy by partner (AOR = 6.2, 95% CI 3.2–12.1)
Gebrezgi	2017	IBCS	Tigray	WHO	422	87 (21)	87 (21)	—	—	—
Fekadu	2018	IBCS	Amhara	WHO	450	264 (59)	145 (32)	34 (8)	260 (58)	House wives (AOR) = 3.43, 95% CI: 1.63, 7.21), no salary of their own (AOR = 3.37, 95% CI: 2.14, 7.95), partners' alcohol use (AOR = 4.59, 95% CI: 1.82, 11.56), women who believed in women's rights to decide to be pregnant (AOR = 1.77, 95% CI: 1.18, 2.89), and women who disobeyed their partner (AOR = 2.36, 95% CI: 1.37, 4.07)
Mohammed	2017	IBCS	Addis Ababa	WHO	210	39 (19)	20 (9.5)	62 (30)	21 (10)	—
Demilash	2015	IBCS	Oromia	SELF	384	100 (26)	75 (20)	54 (14)	76 (20)	—
Bifftu	2017	IBCS	Amhara	WHO	418	106 (25)	34 (8)	10 (2.4)	103 (25)	Low educational status (AOR = 4.59, CI: 1.496, 14.070), rural (AOR = 5.53, CI: 2.311, 13.249), unplanned pregnancy (AOR = 4.34, CI: 2.345, 8.020), and late initiation ANC (AOR = 5.41, CI: 1.493, 19.696)
Kassa	2016	IBCS	SNNP	WHO	216	26 (12)	26 (12)	—	—	—
Gossaye	2003	CBCS	Oromia	WHO	214	164 (77)	164 (77)	—	—	—
Nigussie	2020	IBCS	Amhara	WHO	409	119 (29)	—	—	119 (29)	—

Note: IBCS: institution-based cross-sectional, CBCS: community-based cross-sectional, SNNP: Southern Nation and Nationalities of People.

**Table 2:** Quality of included studies in the analysis (*n* = 26).

Author, year	Quality domain							Overall score
	Selection (max score = 5)				Comparability (max = 2)	Outcome (max = 3)		
	(1) Representativeness of the sample	(2) Sample size	(3) Nonrespondents	(4) Ascertainment of the exposure (risk factor)	(1) Subjects are comparable in different outcome groups	(1) Assessment of outcome	(2) Statistical test	
Ashenafi, 2020	★	★	★	★★	★★	★	★	9
Belay, 2018	★	—	★	★★	★★	★	★	8
Gashaw, 2019	★	★	★	★★	★★	★	★	9
Belay, 2019	★	★	★	★★	★★	★	★	9
Yehunnes, 2019	★	—	★	★★	★★	★	★	8
Azene, 2019	★	—	★	★★	★★	★	★	8
Berhanie, 2019	★	★	★	—	—	★	★	5
G/Selasie, 2020	★	★	★	—	—	★	★	5
Lencha, 2019	★	★	★	★★	★★	★	★	9
Abdurashid, 2013	★	—	★	★★	★★	★	★	8
Tulu, 2019	★	—	★	★★	★★	★	★	8
Adhena, 2020	★	★	★	★★	★★	★	★	9
Tadesse, 2020	★	—	★	★★	★★	★	★	8
Eba, 2014	★	★	★	★★	★★	★	★	9
Laelago, 2017	★	★	★	★★	★★	★	★	9
Mussa, 2020	★	★	★	★★	★★	★	★	9
Abate, 2016	★	—	★	★★	★★	★	★	8
Yimer, 2014	★	★	★	★★	★★	★	★	9
Gebrezgi, 2017	★	★	★	★★	★★	★	★	9
Fekadu, 2018	★	★	★	★★	★★	★	★	9
Mohammed, 2017	★	—	★	★★	★★	★	★	8
Demilash, 2015	★	—	★	—	—	★	★	4
Bifftu, 2017	★	—	★	★★	★★	★	★	8
Kassa, 2016	★	—	★	★★	★★	★	★	8
Gossaye, 2003	★	—	★	★★	★★	★	★	8
Nigussie, 2020	★	—	★	★★	★★	★	★	8

Note: Selection (1) Representativeness of the sample: (a) Truly representative of the average in the target population^∗^ (all subjects or random sampling); (b) somewhat representative of the average in the target population^∗^ (nonrandom sampling); (c) selected group of users; and (d) no description of the sampling strategy. NB: “a” and “b” = ★, “c” and “d” = no ★. (2) Sample size: (a) Justified and satisfactory∗; (b) Not justified. NB: “a” = ★ and “b” = no ★. (3) Nonrespondents: (a) Comparability between respondents and nonrespondent characteristics is established, and the response rate is satisfactory^∗^, (b) the response rate unsatisfactory, or the comparability between respondents and nonrespondents is unsatisfactory, (c) no description of the response rate or the characteristics of the responders and the nonresponders. NB: “a” = ★, “b”, “c” and “d” = no ★. (4) Ascertainment of the exposure/risk factor: Confounding factors are controlled. (a) The study controls for the most important factor (select one)^∗^, (b) The study control for any additional factor^∗^. NB: “a” = 1, “b” = 1. Comparability: The subjects in different outcome groups are comparable, based on the study design (e. g., case or cohort) or analysis. (a) Validated measurement tool. ∗∗, (b) nonvalidated measurement tool, but the tool is available or described,∗ (c) no description of the measurement tool. NB: “a” = ★★, “b” = ★, “c” = no ★. Outcome Assessment of outcome: (a) Independent blind assessment ^∗∗^ (b) Record linkage^∗∗^ (c) Self report ^∗^ (d) No description. NB: “a” and “b” = ★★, “c” = 1 “d” = 0 Statistical test: (a) is clearly described, appropriate, and measurement of association is presented, including confidence intervals and probability level (*p* value) ^∗^ (b) is not appropriate. NB: “a” = ★★, “b” = no ★.

**Table 3:** Subgroup analysis of IPV by study setting, design, sample size, publication year, and study quality.

Subgroup	Overall violence				Physical violence				Sexual violence				Psychological violence			
Subgroup	No. of Sstudies	Prev (95% CI)	*I* ^2^ (%)	*p* value	No. of studies	Prev (95% CI)	*I* ^2^ (%)	*p* value	No. of studies	Prev (95% CI)	*I* ^2^ (%)	*p* value	No. of studies	Prev (95% CI)	*I* ^2^ (%)	*p* value
Study setting																
Oromia	8	51 (35-66)	97.29	≤ 0.001	8	38 (23-54)	97	≤ 0.001	6	21 (11-31)	94.8	≤ 0.001	7	28 (18-42)	94	≤ 0.001
Amhara	7	37 (29-46)	89.13	≤ 0.001	6	20 (22-29)	93	≤ 0.001	5	20 (10-31)	94.4	≤ 0.001	7	32 (24-26)	92	≤ 0.001
SNNP	4	24 (13-35)	92.28	≤ 0.001	4	15 (9-21)	84	≤ 0.001	2	12 (8-15)	0%	0.775	3	20 (14-26)	70.9	0.032
Tigray	4	26 (10-43)	96.52	≤ 0.001	3	17 (12-21)	39	0.193	3	25 (1-50)	98.3	≤ 0.001	2	21 (12-29)	76.2	0.04
Addis Ababa	3	29 (17-40)	89.12	≤ 0.001	3	16 (9-24)	83	0.003	3	25 (19-30)	52	0.124	2	17 (3-32)	93.9	≤ 0.001
Design																
CBCS	5	49 (24-58)	96.7%	≤ 0.001	5	27 (9-46)	98.7%	≤ 0.001	3	12 (9-15)	0%	0.491	4	20 (15-25)	69.5%	0.02
IBCS	21	36 (28-43)	96.6%	≤ 0.001	19	24 (18-29)	94.5%	≤ 0.001	16	23 (17-29)	95.7%	≤ 0.001	17	28 (22-34)	93.7%	≤ 0.001
Sample size																
≥ Median	11	39 (26-51)	97.5%	≤ 0.001	10	25 (16-34)	96.1%	≤ 0.001	9	21 (12-30)	96.5%	≤ 0.001	9	31 (22-39)	94.5%	≤ 0.001
< Median	15	35 (27-43)	95.4%	≤ 0.001	14	24 (16-31)	96%	≤ 0.001	10	21 (16-27)	89.8%	≤ 0.001	12	24 (18-29)	90.6%	≤ 0.001
Publication year																
2003-2015	20	36 (28-44)	96.4%	≤ 0.001	19	23 (17-29)	95%	≤ 0.001	15	22 (15-28)	95.5%	≤ 0.001	17	27 (21-33)	94.5%	≤ 0.001
2016-2020	6	39 (26-51)	95.6%	≤ 0.001	5	29 (11-47)	97%	≤ 0.001	4	18 (14-23)	62.6%	0.046	4	25 (21-28)	31.7%	0.223
Study quality																
Good	23	35 (28-42)	95.8%	≤ 0.001	21	20 (15-25)	92.5%	≤ 0.001	16	20 (15-26)	94.5%	≤ 0.001	18	26 (20-31)	92.2%	≤ 0.001
Fair	3	44 (21-67)	98.2%	≤ 0.001	3	41 (18-63)	98.3%	≤ 0.001	3	26 (10-41)	95.5%	≤ 0.001	3	33 (17-48)	93.7%	≤ 0.001

Note:-95% CI represents the 95% confidence interval for prevalence and *I*^2^ true heterogeneity.

**Table 4:** Factors associated with IPV among pregnant women in Ethiopia.

Factors	Number of studies	Pooled OR (95% CI)	*I* ^2^ (%)	*p* value
No education	5	3.88 (1.48-6.27)	0.0	0.638
Decision-making	4	1.30 (1.03, 1.58)	87.3	≤0.001
No partner education	6	1.10 (0.93, 1.28)	76.6	0.013
Partner alcohol use	11	2.19 (1.66, 2.71)	54.9	0.014
Childhood violence	2	3.14 (1.37, 4.90)	88.8	0.003
Rural	4	2.48 (1.29, 3.67)	0	0.638
Unwanted pregnancy by women	4	1.54 (1.00, 2.08)	70	0.019
Late ANC initiation	3	1.30 (1.15, 1.44)	0	0.374
Partner khat use	3	1.73 (1.42, 2.05)	0	0.593
Unplanned pregnancy	5	1.77 (1.48, 2.05)	24.9	0.255
Unwanted pregnancy by partner	2	1.20 (1.03, 1.36)	79.4	0.027
Family history of DV	2	1.68 (1.14, 2.22)	0	0.650
Attitude toward DV	2	12.92 (6.58, 19.25)	0	0.835

## Data Availability

The data are included in manuscript.

## Abbreviations

CBCS: Community-based cross-sectional
DV: Domestic violence
EDHS: Ethiopian Demographic and Health Survey
IBCS: Institution-based cross-sectional
SNNP: Southern Nation and Nationalities of People
PRISMA: Preferred Reporting Items for Systematic Review and Meta-Analysis
SPSS: Statistical Package for Social Sciences.

## Supplementary Materials

Supplementary Materials PRISMA 2020 Checklist.
